# Pretreatment oral hygiene habits and survival of head and neck squamous cell carcinoma (HNSCC) patients

**DOI:** 10.1186/s12903-016-0185-0

**Published:** 2016-03-11

**Authors:** Juliane Friemel, Ronja Foraita, Kathrin Günther, Mathias Heibeck, Frauke Günther, Maren Pflueger, Hermann Pohlabeln, Thomas Behrens, Jörn Bullerdiek, Rolf Nimzyk, Wolfgang Ahrens

**Affiliations:** Leibniz Institute for Prevention Research and Epidemiology – BIPS, Achterstraße 30, D-28359 Bremen, Germany; Institute for Prevention and Occupational Medicine of the German Social Accident Insurance, Institute of the Ruhr-Universität Bochum (IPA), Bochum, Germany; Center for Human Genetics, University of Bremen (ZHG), Bremen, Germany

**Keywords:** Head and neck squamous cell carcinoma (HNSCC), Dental care, Oral health, Mouthwash use, Tobacco smoking, Alcohol consumption

## Abstract

**Background:**

The survival time of patients with head and neck squamous cell carcinoma (HNSCC) is related to health behavior, such as tobacco smoking and alcohol consumption. Poor oral health (OH), dental care (DC) and the frequent use of mouthwash have been shown to represent independent risk factors for head and neck cancerogenesis, but their impact on the survival of HNSCC patients has not been systematically investigated.

**Methods:**

Two hundred seventy-six incident HNSCC cases recruited for the ARCAGE study were followed through a period of 6–10 years. Interview-based information on wearing of dentures, gum bleeding, teeth brushing, use of floss and dentist visits were grouped into weighted composite scores, i.e. oral health (OH) and dental care (DH). Use of mouthwash was assessed as frequency per day. Also obtained were other types of health behavior, such as smoking, alcohol drinking and diet, appreciated as both confounding and study variables. Endpoints were progression-free survival, overall survival and tumor-specific survival. Prognostic values were estimated using Kaplan-Meier analysis and Cox proportional hazards regression models.

**Results:**

A good dental care score, summarizing annual dental visits, daily teeth cleaning and use of floss was associated with longer overall survival time (*p* = .001). The results of the Cox regression models similarly suggested a higher risk of tumor progression and shortened overall survival in patients with poor dental care, but the results lost their statistical significance after other types of health behavior had been controlled for. Frequent use of mouthwash (≥ 2 times/day) significantly increased the risk of tumor-specific death (HR = 2.26; CI = 1.19–4.32). Alcohol consumption and tobacco smoking were dose-dependently associated with tumor progression and shorter overall survival.

**Conclusion:**

Frequent mouthwash use of ≥ 2 times/day seems to elevate the risk of tumor-specific death in HNSCC patients. Good dental care scores are associated with longer overall survival.

**Electronic supplementary material:**

The online version of this article (doi:10.1186/s12903-016-0185-0) contains supplementary material, which is available to authorized users.

## Background

Head and neck squamous cell carcinomas (HNSCC) account for 90 % of all head and neck cancers. Locations include the lip, oral cavity, pharynx and larynx. HNSCC is the 6th most common cancer worldwide [1]. It accounts for approximately 100,000 cases diagnosed annually in the European Union, and the incidence is increasing [2–4].

Tobacco smoking, alcohol consumption, HPV infection and socioeconomic factors are established risk factors for cancer development in the head and neck region [5–8]. Dietary factors, such as the consumption of vegetables and fruits, have been described as having a protective effect [9–13]. A growing body of evidence suggests that a lack of dental care and poor oral health have to be considered as independent risk factors for HNSCC development [14–17].

Many of the established risk factors for cancerogenesis in the head and neck region also have a prognostic value, as has been demonstrated in follow-up studies of patients with incident HNSCC [5, 18–20]. Studies largely agree about the effect of smoking and HPV status on survival. Alcohol consumption and diet as independent variables for overall or progression-free survival are controversial [18, 20–22].

In contrast, the potential effect of dental care, oral health and mouthwash use on survival in head and neck cancer patients have not been systematically investigated. Therefore, this study was designed to determine whether oral health, dental care and mouthwash use have prognostic value for the survival of HNSCC patients. Two novel, recently published scores for assessing dental care and oral health status [14] were used in addition to the evaluation of other types of health behavior, such as smoking, drinking, and vegetable consumption. All lifestyle variables were tested for their effect on overall survival, tumor progression and tumor-specific survival of HNSCC patients.

## Methods

### Study population

Two hundred seventy-six incident cases (Germany) with pathologically confirmed diagnosis of head and neck squamous cell carcinoma (HNSCC) were originally recruited in a multicenter study (ARCAGE) [7]. Locations comprised the oral cavity (C01-C06), tonsils (C09), pharynx (C10-C13) and larynx (C32). Cases with in-situ carcinoma, esophagus cancer, cancer of the vermillion border, the paranasal sinuses and salivary glands were not included.

Information about risk factor exposure, including oral hygiene behavior, was assessed through standardized computer assisted personal interviews during the ARCAGE study. All subjects signed a form ensuring informed consent, ethical approval was given by the IARC ethical review board. For the follow-up study, pathological data were collected from cancer registries supplemented by pathology reports. The tumor stage was assessed according to UICC (Tumor, Node, Metastasis [TNM] stage I-IV). Cancer treatment such as radiotherapy, chemotherapy and surgery was abstracted from clinical records. The average follow-up time covered a period of 8 years (range 6–10). Survival data, such as cause of death or occurrence of metastasis or relapse (tumor progression), were obtained from local health departments, medical practitioners and the Bremen mortality index [23].

### Health behavior variables

Interview-based assessment of oral hygiene habits and other health behavior variables was performed during hospitalization of HNSCC patients. The median time point of the Interview was 6 days after primary tumor treatment, interview questions referred to the time period of one year or more prior to diagnosis. Oral health and dental care were represented by weighted composite scores, which were constructed *a priori* based on repeatedly reported variables associated with HNSCC [14, 15, 24]. As shown in Additional file 1: Table S1, the oral health (OH) score involved a 0–7 scale and the dental care (DC) score a 0–8 scale, with higher numbers indicating poorer oral health or dental care. In brief, each variable summarized the information on three indicators. The oral health score accounted for wearing dentures, age at which patient started to wear dentures and the frequency of gum bleeding. Gum bleeding usually occurs during chronic periodontitis and can contribute to cancerogenesis [25–27]. Individuals not sufficiently taking care of their teeth and gum tend to wear dentures earlier in life. Missing teeth alone have been shown increase HNSCC risk [15]. Poor fitting dentures are associated with a 4-fold increased risk for oral cancer [24]. The dental care score comprised number of dentist visits per year, frequency of teeth cleaning and use of floss. Use of mouthwash was measured as frequency per day, regardless of its formulation. Composite scores and mouthwash were grouped into two categories. Here, an OH score of 6–7, a DC score of 3–8 and mouthwash use ≥ 2/d served as the exposure, ensuring that the categories included a meaningful number of subjects. The other categories were defined as reference. In a sensitivity analysis, hazard ratios for oral hygiene habits were estimated using different types of categorization in order to minimize the probability for misleading results (Additional file 2: Table S2).

Smoking behavior was operationalized by pack-years (20 cigarettes per day multiplied by number of years), smoking frequency (cigarettes per day) and smoking duration (years). “Smokers” were defined as individuals smoking cigarettes, cigars, pipe, or any tobacco product at least once per week for a year [7]. Participants who had stopped smoking less than 1 year ago or by the time of tumor diagnosis were classified as current smokers.

Alcohol consumption was operationalized by drink-years (drinks per day multiplied by number of years), drinking frequency (number of drinks per day) and drinking duration (years of alcohol consumption). The definition of one alcoholic drink equivalent was 18 ml of pure alcohol, which generally corresponds to 330 ml of beer, 150 ml of wine and 36 ml of hard liquor [7]. Participants who had stopped drinking alcohol less than 1 year ago or by the time of tumor diagnosis were classified as current drinkers. Cumulative exposure variables as pack-years and drink-years reflect the product of intensity and duration of the exposure and are good predictors for many exposure-response relationships [28]. Diet was operationalized by fruit and vegetable consumption (consumption frequency per week, type of fruit). The patients’ education was represented by years of school education (< 10 years [≤ ISCED level 2], ≥ 10 years).

### HPV assessment

Human papilloma virus (HPV) DNA was detected using PCR methods as established before [29]. 150 ng tumor DNA was extracted from formalin-fixed, paraffin embedded tissues from primary tumor sites (Quiagen, Hilden, Germany) and eluted in 25 μl molecular grade water. Incubation with 0.5 U Uracil-DNA-Glycosylase (UNG) for 5 min at 20° was followed by thermal inactivation of UNG for 2 min at 95 °C. The PCR was performed with an initial denaturation (30 s at 98 °C), followed by denaturation (6 s at 98 °C), annealing (15 s at 40 °C) and elongation (5 s at 72 °C) including a final elongation step for 3 min at 72 °C.

To verify that HPV high risk types 16 and 18 were detected, the following primers were used: HPV16- ATATAAGGGGTCGGTGGACCG, GCAATGTAGGTGTATCTCCATGC and HPV18- AAGGATGCTGCACCGGCTGAA, CACGCACACGCTTGGCAGGTTT). The PCR (PCR core plus, Roche, Switzerland) was performed following to the manufacturer’s protocol, using incubation with 0.5 U Uracil-DNA-Glycosylase (UNG) for 5 min at 20 °C and consecutive thermal inactivation of UNG for 2 min at 95 °C. The PCR (35 amplification cycles) was performed with an initial denaturation (30 s at 98 °C), followed by denaturation (20 s at 98 °C), annealing (15 s at 55 °C) and elongation (20 s at 72 °C) including a final elongation step for 3 min at 72 °C.

### Outcomes

The study outcomes were overall survival, tumor progression free survival and tumor-specific survival. Overall survival was defined as time between tumor diagnosis and death or end of the study. Tumor progression was defined as time between diagnosis and occurrence of metastasis or tumor relapse. Tumor-specific survival was defined as time between HNSCC diagnosis to death related to the primary tumor or to the end of the study. Progression-free survival was used if patients stayed alive without tumor relapse during the observational period. If no further visits to health departments/medical practitioners were documented (loss to follow up), the last day of a documented visit to a health department/medical practitioner was the time point of censoring. In the setting of tumor-specific survival, those patients were censored who had not died from their HNSCC. Ethical approval was given by the institutional review board of the Medical Association Bremen (44-110-10.10/4).

### Statistical methods

Curves of overall survival were obtained by the Kaplan-Meier method. Differences in survival between groups were determined by the log-rank test. A Cox proportional hazards regression model was applied to assess the association between lifestyle variables and the endpoints overall survival, tumor progression and tumor-specific survival, considering a significance level of α = 5 %. The Cox regression model was adjusted for sex, age, tumor stage according to UICC criteria (stage I-IV and missing), tumor site (oral cavity (C01-C06), tonsils (C09), pharynx (C10-C13) and larynx (C32), treatment (radiotherapy, surgery and chemotherapy measured as yes and no/unknown responses), education (more or less than ten years of education), smoking (pack-years), alcohol consumption (drink-years), vegetable and fruit consumption (times per week) and HPV-16/18-infection status of the tumor, which are established influencing factors for HNSCC patient survival [8, 20, 30] . Smoking and alcohol consumption were examined as continuous variables after natural logarithmic transformation. Missing values were categorized as separate category and included in the analyses to make use of the entire sample. Statistical analyses were performed using the statistical software package SAS 9.3.

## Results

### Patient characteristics

Table 1 displays the patient characteristics of 276 incident cases of head and neck squamous cell carcinoma (HNSCC). The male–female ratio was 5:1, the mean age was 58 years (SD ± 9). Almost 70 % of the patients had received less than 10 years of school education. The majority of patients had tumors of the oral cavity (*n* = 89, 32 %), the larynx (*n* = 76, 28 %) and of the pharynx (*n* = 72, 26 %). Less frequent were tumors of the tonsils (*n* = 39, 14 %). Most patients with available information on tumor stage had stage IV tumors (*n* = 125, 45 %). Tumor stages I/II/III were nearly uniformly distributed (*n* = 22/33/27). If one of the mandatory grading parameters T (primary tumor), N (regional lymph nodes) and M (distant metastasis) was not reported, tumor classification (UICC) could not been derived and was set to missing. The majority of 228 patients (83 %) underwent surgery; 184 patients (67 %) had radiotherapy as single or adjuvant treatment and in 107 patients (39 %) chemotherapy was administered. Overall, 24 of 191 patients (12.6 %) with available HPV test results had HPV-related squamous cell carcinoma. HPV status was set to missing if tumor tissue was not collected during the ARCAGE study. In agreement with previous studies, the incidence of HPV association was low among patients with HNSCC of the oral cavity (8.2 %). Conversely, in 40.7 % of patients with tonsillar HNSCC an exposure to high risk HPV could be detected. HNSCC of the larynx in our collective had the lowest HPV incidence (3.8 %), which might be due to the small number of samples and the high number of missing values of the HPV status.

**Table 1:** Demography and clinico-pathological data of patients with head and neck squamous cell carcinoma (HNSCC)

	Men	Women	All
	(*N* = 230)	(*N* = 46)	(*N* = 276)
Demography			
Age (years)			
Mean (SD)	58.2 (8.5)	57.0 (11.4)	58.0 (9.0)
Median (IQR)	58.0 (12.0)	58.0 (15.0)	58.0 (12.0)
Range	41–77	32–77	32–77
Education (years)			
< 10	164 (73.3 %)	25 (54.4 %)	189 (68.5 %)
≥ 10	66 (28.7 %)	21 (45.6 %)	87 (31.5 %)
Clinico-pathological characteristics			
Stage (UICC^a^)			
I	19 (8.3 %)	3 (6.5 %)	22 (8.0 %)
II	28 (12.2 %)	5 (10.9 %)	33 (12.0 %)
III	19 (8.3 %)	8 (17.4 %)	27 (9.8 %)
IV	106 (46.1 %)	19 (41.3 %)	125 (45.3 %)
Missing	58 (25.2 %)	11 (23.9 %)	69 (25.0 %)
Tumor site			
C01-C06 (Oral cavity)	70 (30.4 %)	19 (41.3 %)	89 (32.3 %)
C09 (Tonsils)	34 (14.8 %)	5 (10.9 %)	39 (14.1 %)
C10-C13 (Pharynx)	56 (24.4 %)	16 (34.8 %)	72 (26.1 %)
C32 (Larynx)	70 (30.4 %)	6 (13.0 %)	76 (27.5 %)
Radiotherapy			
No/unknown	78 (33.9 %)	14 (30.4 %)	92 (33.3 %)
Yes	152 (66.1 %)	32 (69.6 %)	184 (66.7 %)
Surgery			
No/unknown	38 (16.5 %)	10 (21.7 %)	48 (17.4 %)
Yes	192 (83.5 %)	36 (78.3 %)	228 (82.6 %)
Chemotherapy			
No/unknown	145 (63.0 %)	24 (52.2 %)	169 (61.2 %)
Yes	85 (37.0 %)	22 (47.8 %)	107 (38.8 %)
HPV status (16, 18)			
Negative	142 (61.7 %)	25 (54.4 %)	167 (60.5 %)
Positive	19 (8.3 %)	5 (10.9 %)	24 (8.7 %)
Missing	69 (30.0 %)	16 (34.8 %)	85 (30.8 %)

^a^Union of International Cancer Classification

### Dental care and oral health

Table 2 illustrates the distributions of the composite variables dental care and oral health among potentially confounding health behaviors. 159 patients (58 %) reported good dental care as defined (DC ≤ 2, Table 2). Poor dental care (score of 3–6) was seen in 38 patients (14 %). None of the patients had a poorer dental care than score 6. Thirty patients (11 %) stated that they used mouthwash more than once a day. Indicators of poor oral health (wearing of dentures, gum bleeding) were present in 62 patients (22 %). In contrast, 194 patients claimed good oral health (70 %). Values for DC were missing in 79 cases and for OH in 20 cases.

**Table 2:** Health behavior of patients with head and neck squamous cell carcinoma (HNSCC)

	Men	Women	All
	(*N* = 230)	(*N* = 46)	(*N* = 276)
Dental Care^a^			
0–2 (good)	130 (56.5 %)	29 (63.0 %)	159 (57.6 %)
3–6 (bad)	36 (15.7 %)	2 (4.4 %)	38 (13.8 %)
Missing	64 (27.8 %)	15 (32.6 %)	79 (28.6 %)
Oral Health^a^			
0–5 (good)	165 (71.7 %)	29 (63.0 %)	194 (70.3 %)
6–7 (bad)	47 (20.4 %)	15 (32.6 %)	62 (22.5 %)
Missing	18 (7.8 %)	2 (4.4 %)	20 (7.3 %)
Mouthwash (times per day)			
< 2	202 (87.8 %)	42 (91.3 %)	244 (88.4 %)
≥ 2	26 (11.3 %)	4 (8.7 %)	30 (10.9 %)
Missing	2 (0.9 %)	0 (0.0 %)	2 (0.7 %)
Pack-years			
Mean (SD)	44.1 (26.2)	27.7 (20.4)	41.4 (26.0)
Median (IQR)	40.0 (26.1)	25.7 (19.2)	36.8 (27.6)
Range	0–203	0–92	0–203
Missing	3	0	3
Smoking frequency (per day)			
Mean (SD)	23.6 (12.1)	16.3 (10.5)	22.4 22.4 (12.1)
Median (IQR)	20.1 (11.0)	15.9 (12.1)	20.0 (11.2)
Range	0–104	0–44	0–104
Missing	3	0	3
Smoking duration (years)			
Mean (SD)	36.2 (11.8)	31.0 (14.3)	35.3 (12.4)
Median (IQR)	37.5 (13.0)	34.0 (11.0)	37.0 (12.5)
Range	0–60	0–49	0–60
Missing	2	0	2
Drink-years			
Mean (SD)	87.5 (101.7)	35.1 (92.5)	78.7 (101.9)
Median (IQR)	46.5 (101.8)	13.4 (26.7)	41.0 (83.5)
Range	0–573	0–621	0–621
Missing	3	0	3
Drinking frequency (per day)			
Mean (SD)	2.6 (3.1)	1.1 (2.9)	2.3 (3.1)
Median (IQR)	1.4 (2.8)	0.4 (0.8)	1.1 (2.7)
Range	0–16	0–19	0–19
Missing	3	0	3
Drinking duration (years)			
Mean (SD)	36.5 (11.0)	28.3 (15.3)	35.1 (12.2)
Median (IQR)	36.5 (15.0)	31.0 (20.0)	36.0 (15.0)
Range	0–61	0–53	0–61
Missing	2	0	2
Vegetable consumption			
Mean (SD)	4.4 (2.6)	5.1 (3.2)	4.5 (2.7)
Median (IQR)	4.0 (5.0)	4.0 (4.0)	4.0 (4.7)
Range	0–14	1–14	0–14
Missing	9	3	12

^a^The dental care score comprised number of dentist visits per year, frequency of teeth cleaning and use of floss. The oral health score accounted for wearing dentures, age at which patient started to wear dentures and frequency of gum bleeding. Composite scores and mouthwash were grouped into two categories. Here, an OH score of 6–7, a DC score of 3–6 and mouthwash use ≥ 2/d served as the exposure, ensuring that the categories included a meaningful number of subjects

Looking at the distribution of the other types of health behavior, such as smoking and alcohol consumption, it is noteworthy that the mean number of pack-years within the patient cohort was 41 (median: 37 pack-years). The mean number of drink-years was 79 (median: 41 drink-years). The mean frequency of vegetable and fruit servings was 5 per week.

Missing data in health behavior variables resulted from a missing response for the respective item in the questionnaire.

### Influence of dental care, oral health and mouthwash on different survival endpoints

**Fig. 1:**
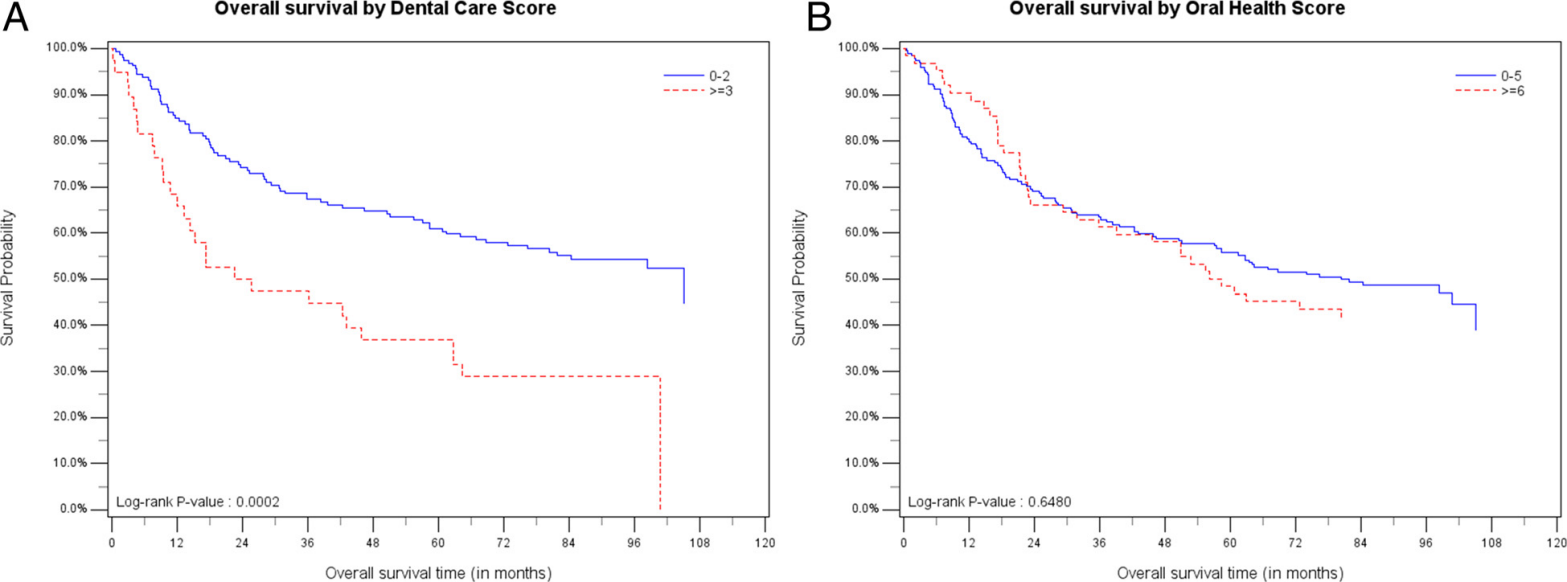
Kaplan-Meier curves showing the differences in overall survival time between patients with good dental care/oral health (=blue) and poor dental care/oral health (= red). The difference in median survival time is 81 months for dental care (**a**) and 23 months for oral health (**b**)

A good dental care score, represented by annual dental visits, daily teeth cleaning and use of floss, showed a significant benefit looking at the overall survival time in a Kaplan-Meier analysis (Fig. 1). The difference in median survival time between patients with good vs poor dental care was 81 months (*p* < 0.001). Hazard ratios of the Cox regression model (Table 3) imply that lack of dental care might contribute to the risk of earlier death or tumor progression in HNSCC patients (1.5-fold), but the effect was not statistically significant. Looking at the endpoint of tumor-specific survival, frequent mouthwash use, i.e. more than once a day, showed a significant effect (HR = 2.26; CI 95 % = 1.19–4.32), indicating that patients with frequent mouthwash use are more likely to die because of their tumor disease. Overall survival and tumor progression were also negatively influenced by frequent mouthwash use, but no statistically significant effect could be demonstrated (Table 3). Frequency of gum bleeding and wearing dentures (oral health score) did not show an effect on HNSCC prognosis.

**Table 3:** Hazard ratios (HR) for the influence of health behavior variables on tumor survival in HNSCC patients

Variable	HR overall survival^a^		HR progression free survival^a^		HR tumorspecific survival^a^	
	(*n* = 263)	95 % CI	(*n* = 254)	95 % CI	(*n* = 261)	95 % CI
Dental care score^b^						
Score 0–2	1.00	Ref.	1.00	Ref.	1.00	Ref.
Score 3–6 (poor care)	1.30	0.78–2.15	1.48	0.89–2.45	1.51	0.79–2.88
Mouthwash use^d^						
Mouthwash < 2 times/day	1.00	Ref.	1.00	Ref.	1.00	Ref.
Mouthwash ≥ 2 times/day	1.32	0.76–2.31	1.47	0.88–2.47	**2.26**	1.19–4.32
Oral health score^c^						
Oral health 0–5	1.00	Ref.	1.00	Ref.	1.00	Ref.
Oral health 6–7 (poor health)	1.05	0.69–1.60	1.11	0.74–1.67	0.85	0.47–1.54
Smoking *log[pack-years + 1]*						
10 pack-years	**2.05**	**1.17–3.61**	**1.89**	**1.11–3.24**	1.72	0.83–3.56
20 pack-years	**2.46**	**1.21–4.97**	**2.22**	**1.14–4.34**	1.97	0.79–4.89
Smoking freq *log[cigarettes + 1]*						
10 cigarettes/day	**2.30**	**1.17–4.52**	**2.04**	**1.07–3.89**	2.06	0.83–5.09
20 cigarettes/day	**2.83**	**1.22–6.58**	**2.44**	**1.09–5.46**	2.47	0.80–7.64
Drinking (drink-years) *log[drinkyears + 1]*						
10 drink-years	**1.59**	**1.14–2.24**	**1.53**	**1.10–2.11**	**1.90**	**1.21–2.99**
20 drink-years	**1.79**	**1.17–2.74**	**1.70**	**1.13–2.55**	**2.23**	**1.27–3.93**
Drinking freq *log[drinks + 1]*						
2 drinks/day	**1.47**	**1.13–1.90**	**1.52**	**1.18–1.97**	**1.52**	**1.08–2.12**
5 drinks/day	**1.87**	**1.22–2.87**	**1.99**	**1.31–3.04**	**1.97**	**1.14–3.41**

Bold numbers indicate statistically significant log rank tests and HR

*Abbreviations: freq* frequency, *log* logarithmic transformation, *Ref* reference

^a^Hazard ratios: Cox regression model adjusted for age, sex, tumor site, tumor stage, treatment, education, smoking, alcohol drinking, HPV status. Hazard ratios describe the risk of death, tumor progression or tumor specific death within the observational period; ^b^includes information on brushing teeth, use of floss, dentist visits ^c^includes information on wearing dentures, age at wearing dentures, gum bleeding; ^d^independent variable

A strong, dose-dependent association between survival time and lifestyle variables was seen for smoking and drinking habits. Under the assumption of proportional hazards, the risk of tumor recurrence or death (overall survival, HR = 2.83; CI 95 % = 1.22–6.58) was increased nearly threefold for patients who smoked 20 cigarettes per day. A drinking frequency of 5 drinks/day showed a stronger adverse effect on survival (overall survival, HR = 1.87; CI 95 % = 1.22–2.87) than an accumulation of 10 drink-years (overall survival, HR =1.59; CI 95 % = 1.14–2.24).

Apart from mouthwash use, tumor-specific survival was significantly reduced by a drinking frequency of 2 drinks/day (HR = 1.52; CI 95 % = 1.08–2.12). The number of vegetable or fruit servings per week had no influence on patient survival (data not shown).

## Discussion

Our study demonstrates that annual dental visits, daily teeth cleaning and use of floss (low dental care score) are associated with longer overall survival in patients with head and neck squamous cell carcinoma (HNSCC). Frequent mouthwash use (≥ 2 times/day) was associated with a twofold increased risk of tumor-specific death in HNSCC patients after other lifestyle factors had been controlled for. Hazard ratios for the association between poor dental care and the risk of death or tumor progression were consistently elevated over all endpoints, but not statistically significant. Smoking and alcohol consumption were dose-dependently associated with a maximally threefold higher risk of tumor progression and shortened overall survival.

Poor oral hygiene habits are independent risk factors for the development of HNSCC in multiple studies [14–16, 31, 32], but the operationalization of habits and adjustment for confounder is not homogenous at all. The ARCAGE study (2002–2005) [14], to date the largest multicenter case–control study included 1963 cancer patients’ vs 1993 control subjects. Novel composite scores for dental care and oral health were developed as the summary of previously reported criteria like wearing of dentures, frequency of gum bleeding, teeth brushing, use of brush/toothpaste/floss and regular dentist visits [15, 16, 24]. Although not validated by inspections of the mouth and a possible recall bias of the interview- based assessment, Ahrens et al. observed consistent associations between the scores and upper-aerodigestive tract cancers across different subsites. Using these composite weighted scores, we first evaluated the prognostic relevance of oral hygiene habits on survival in 276 HNSCC patients. Patients with good oral hygiene habits lived significantly longer, but the results of the Cox regression analysis controlling for established survival predictors like age, sex, tumor stage, tumor site, treatment, education, HPV status, smoking and alcohol consumption, were insignificant. Nevertheless, elevated hazard ratios suggest that good dental care might positively influence survival in HNSCC patients, but the informative value is limited by the small sample size of our study. Gum bleeding and wearing of dentures (parameters of the oral health score) did not have an effect on tumor survival in univariate or multivariate analysis.

That frequent mouthwash use significantly increased the risk of tumor-specific death, support the hypothesis that mouthwash contributes to the development and evolution of HNSCC. Several studies speculate about an association of mouthwash and head and neck cancer development [32–34]. Earlier works found that especially alcohol containing mouthwashes elevate the risk of oral and pharyngeal cancer [33]. A more recent study by Eliot et al. [16] showed that both alcoholic and non-alcoholic mouthwashes are a risk for HNSCC when used at least once per day. In contrast, a quantitative meta-analysis by Gandini et al. [35] revealed no significant associations between mouthwash and oral cancer risk. Within the ARCAGE cohort, there was a 3.5-fold risk increase of head and neck cancer development (sites included mouth, oropharynx, hypopharynx, larynx and esophagus) in individuals using alcoholic or non-alcoholic mouthwashes three or more times daily compared to never-users [14]. Like in our follow up study using ARCAGE data, unfortunately no discrimination could be made between alcoholic and non-alcoholic mouthwashes. Similarly, the overall lifetime exposure to mouthwash was not assessed. Nevertheless, the impact of mouthwash use on HNSCC risk could be demonstrated, whilst confounding such as tobacco use, alcohol consumption and HPV status has been controlled for through logistic regression models. Also it has been suggested that mouthwash use might simply be a “masking” behavior to get rid of the smokers’ breath [36]. In a study investigating oral hygiene habits of smokers and non-smokers, the frequency of mouthwash use did not differ between the two groups [37].

In agreement with other studies we observed that smoking and alcohol consumption were dose-dependently associated with a higher risk of shortened overall survival of HNSCC patients. A study by Mayne and colleagues [21] on 264 patients showed a significant influence of alcohol and tobacco consumption on survival in early stage cancer in the oral cavity, the pharynx and the larynx. In the prospective part of the study, Mayne et al. stated that the risk of dying was increased twofold in patients who continued to drink alcohol. Continued smoking did not increase the risk of dying. A study by Duffy and colleagues [20] investigating the influence of various (pretreatment) health behaviors, found that smoking was the strongest independent predictor of survival among 504 head and neck cancer patients, but not alcohol consumption. In our analysis of the influence of alcohol consumption, we calculated that the risk of tumor progression associated with drinking frequencies of 2 drinks per day was increased 1.5-fold. While we modeled alcohol consumption and smoking as log-transformed continuous variables, many studies use categorized variables, which may increase the probability of false positive results [38].

Second cancers following oral and pharyngeal cancers as endpoint with respect to the smoking and drinking behavior were investigated by Day et al. [18]. This follow-up study on 1090 patients recruited in a population-based case–control study showed that the risk of a second aero-digestive tract cancer increased fourfold among smokers who had smoked more than 40 years compared to smokers who had smoked less than 20 years at the time of diagnosis. Among alcohol consuming individuals, the risk of local tumor recurrence was increased threefold in the head and neck region [18]. In our study we observed a 2.2-fold increased risk of tumor progression (observed events included tumor recurrences) associated with 20 pack-years and a 1.7-fold increased risk associated with 20 drink-years.

In contrast to some of the mentioned follow-up studies, we did not obtain follow-up information on health behavior through second interviews because many patients had died at the time of follow up assessment. It is worthy of note that significant survival prediction, as documented in many studies [20, 21, 39], mostly refer to pretreatment health behavior. The stronger effect of pretreatment compared to posttreatment lifestyle behavior is also supported by Franceschi and colleagues [22]. Their study, including 754 head and neck cancer patients, showed that ceasing drinking at the time of cancer diagnosis does not have a clearly favorable effect on survival. Marron et al. demonstrated that quitting alcohol drinking reduces the risk for HNSCC development not before 20 years of abstinence, while smoking cessation reduced the risk after 1–4 years [40]. The favorable effect of smoking cessation on recurrence rates and tumor survival has been demonstrated by many studies summarized in a comprehensive review by van Imhof et al. [41].

## Conclusion

In conclusion, frequent mouthwash might be an independent prognostic factor for tumor-specific survival. Good dental care, comprising annual dental visits, daily teeth cleaning and use of floss is associated with longer overall survival, but the trend could not be proven by Cox regression analysis. Alcohol consumption and tobacco smoking were dose-dependently associated with tumor progression and shorter overall survival.

## Additional files

Additional file 1: Table S1. Operationalizing of the composite score. (DOCX 16 kb) Additional file 2: Table S2. Variants of categorization of the study variable dental care and oral health. (DOCX 20 kb)

## Abbreviations

ARCAGE: Alcohol related cancers and genetic susceptibility in Europe
HNSCC: head and neck squamous cell carcinoma
HPV: Human papilloma virus
TNM: Tumor Node Metastasis
