# Supplementary material for: Pretreatment oral hygiene habits and survival of head and neck squamous cell carcinoma (HNSCC) patients
Source: BMC Oral Health. 2016 Mar 11;16:33. doi: 10.1186/s12903-016-0185-0 (PMC4788953; doi:10.1186/s12903-016-0185-0)
Supplement: Additional file 1: Table S1. — Operationalizing of the composite score. (DOCX 16 kb) [file 12903_2016_185_MOESM1_ESM.docx]

**Table S1: Operationalizing of the composite score**

| **Oral health** | **Score** | **Specification** |
| --- | --- | --- |
| Wearing of dentures | **0** | no denture |
|  | **1** | partial denture in upper or lower jaw |
|  | **2** | partial denture in both jaws |
|  | **3** | complete denture in upper or lower jaw |
|  | **4** | complete denture in both jaws |
| Age at starting to wear dentures | **0** | no denture |
|  | **1** | denture at age 55 or older |
|  | **2** | denture at age 35-54 years |
|  | **3** | denture at age below 35 years |
| Frequency of gum bleeding from brushing teeth | **0*** | sometimes or never |
|  | **1** | always or almost always |
| **Dental care** | **Score** | **Specification** |
| Frequency of teeth cleaning | **0** | at least twice/day |
|  | **1** | once/day |
|  | **2** | 1-4 times/week |
|  | **3** | less often or never |
| Use of toothbrush, toothpaste or dental floss | **0** | two or three of these |
|  | **1** | only one of these three |
|  | **2** | none of these |
| Frequency of dentist visits | **0** | at least once a year |
|  | **1** | every 2-5 years |
|  | **2** | less than every 5 years |
|  | **3** | never |
| *score 0 was always applied in patients with complete dentures in both jaws | | |
