# Supplementary material for: Pretreatment oral hygiene habits and survival of head and neck squamous cell carcinoma (HNSCC) patients
Source: BMC Oral Health. 2016 Mar 11;16:33. doi: 10.1186/s12903-016-0185-0 (PMC4788953; doi:10.1186/s12903-016-0185-0)
Supplement: Additional file 2: Table S2. — Variants of categorization of the study variable dental care and oral health. (DOCX 20 kb) [file 12903_2016_185_MOESM2_ESM.docx]

| **Variable** | **HR overall survival** | **95% CI Lower Upper** | | **HR progression free survival** | **95% CI Lower Upper** | | **HR tumor-specific survival** | **95% CI Lower Upper** | |
| --- | --- | --- | --- | --- | --- | --- | --- | --- | --- |
| **Dentalcare** |  |  |  |  |  |  |  |  |  |
| Score 0 | 1 | Ref. | | 1 | Ref. | | 1 | Ref. | |
| *Categorisation 1* |  |  |  |  |  |  |  |  |  |
| Score 1,2 | 0.99 | 0.59 | 1.65 | 0.96 | 0.58 | 1.58 | 1.05 | 0.51 | 2.16 |
| Score 3-6 | 1.28 | 0.70 | 2.37 | 1.44 | 0.79 | 2.62 | 1.56 | 0.69 | 3.53 |
| *Categorisation 2* |  |  |  |  |  |  |  |  |  |
| Score 1-3 | 1.09 | 0.67 | 1.78 | 1.11 | 0.69 | 1.79 | 1.26 | 0.64 | 2.49 |
| Score 4-6 | 0.88 | 0.38 | 2.05 | 0.77 | 0.33 | 1.78 | 0.62 | 0.18 | 2.19 |
| **Oralhealth** |  |  |  |  |  |  |  |  |  |
| Score 0 | 1 | Ref. | | 1 | Ref. | | 1 | Ref. | |
| *Categorisation 1* |  |  |  |  |  |  |  |  |  |
| Score 1-5 | 1.51 | 0.98 | 2.32 | 1.46 | 0.96 | 2.23 | 1.60 | 0.91 | 2.80 |
| Score >=6 | 1.34 | 0.81 | 2.21 | 1.40 | 0.86 | 2.27 | 1.12 | 0.56 | 2.21 |
| *Categorisation 2* |  |  |  |  |  |  |  |  |  |
| Score 1-6 | 1.47 | 0.98 | 2.21 | 1.45 | 0.97 | 2.17 | 1.43 | 0.84 | 2.44 |
| Score 7 | 1.12 | 0.46 | 2.73 | 1.28 | 0.57 | 2.85 | 1.29 | 0.42 | 3.96 |
| **Mouthwash** |  |  |  |  |  |  |  |  |  |
| Never | 1 | Ref. | | 1 | Ref. | | 1 | Ref. | |
| *Categorisation 1* |  |  |  |  |  |  |  |  |  |
| <2 times /day | 1.07 | 0.73 | 1.56 | 1.08 | 0.74 | 1.57 | 1.33 | 0.80 | 2.21 |
| >= 2 times /day | 1.32 | 0.74 | 2.36 | 1.48 | 0.87 | 2.53 | **2.44** | **1.24** | **4.81** |
| *Categorisation 2* |  |  |  |  |  |  |  |  |  |
| <3 times /day | 1.06 | 0.74 | 1.53 | 1.12 | 0.79 | 1.60 | 1.45 | 0.90 | 2.34 |
| >= 3 times /day | **2.67** | **1.08** | **6.61** | 2.05 | 0.85 | 4.99 | **5.62** | **1.78** | **17.7** |

**Table S2: Variants of categorization of the study variable dental care and oral health**
